# Switching to remimazolam followed by flumazenil may be a promising combination for deep extubation

**DOI:** 10.1186/s40981-023-00643-7

**Published:** 2023-08-11

**Authors:** Hideharu Araki, Satoki Inoue

**Affiliations:** 1 Department of Anesthesiology, Minamisoma Municipal General Hospital, Minamisoma, Japan; 2 Department of Anesthesiology, Fukushima Medical University School of Medicine, Fukushima City, Fukushima, 960-1295 Japan

To the Editor,

Tracheal extubation under deep anesthesia, known as deep extubation, has widely been used as a technique that enables smooth emergence from anesthesia. However, anesthesiologists are concerned about potential respiratory complications that this technique may cause [1]. In addition, it has been reported that there is a need for prolonged observation in the operating room or postanesthesia care units, although the complication rate has been relatively low [2]. We here report a case of successful deep extubation in a patient under remimazolam-based anesthesia.

A 60-year-old man (170 cm and 90 kg) with bronchial asthma was scheduled for laparoscopic colectomy. He had had several mild asthma attacks, despite having been treated with inhaled corticosteroids, long-acting beta-agonists, long-acting muscarinic agonists, oral steroids, leukotriene receptor antagonists, and theophylline. He had not received biopharmaceutical agents for personal reasons. Deep extubation was planned to prevent airway irritation. Anesthesia was induced with remimazolam 3 mg/kg/h (total 12 mg) and remifentanil 0.15 μg/kg/min. After obtaining loss of consciousness, rocuronium 60 mg was administered, and tracheal intubation was performed. Subsequently, remimazolam was switched to 1.5–2% sevoflurane, and anesthesia was adjusted according to the patient state index (PSI) between 25 and 50. Neuromuscular blockade was monitored, and post-tetanic count (PTC) < 10 was maintained. Analgesia was provided by epidural anesthesia and remifentanil. After completion of the laparoscopic procedures lasting about 3 h, sevoflurane was switched to remimazolam. In short, administration of sevoflurane was stopped, and the same induction dose of remimazolam was administered. Following the switching of the sedative agent, remimazolam 0.8 mg/kg/h was administered until the skin closure was completed. Administration of rocuronium was ceased at the end of surgery. After complete reversal of muscular relaxation by sugammadex 400 mg (train of four ratio > 95%), tracheal extubation was performed under sustained deep anesthesia and was completed smoothly without inducing asthma attack. At that time, the PSI showed around 50. Immediately after extubation, flumazenil 0.9 mg was administered. Manual facemask ventilation to assist his spontaneous ventilation was performed until he regained consciousness. Soon after (1–1.5 min later), he became fully awake and responded to any verbal commands without any complaint. His postoperative course was uneventful.

Tracheal extubation in unconscious patients can increase the risk of hypoxemia and prolong recovery time, which may make some anesthesiologists hesitate to perform deep extubation [1, 2]. The best advantages of remimazolam for deep extubation are that its effects are reversed by flumazenil, and it has an ultrashort duration of action [3]. These properties of remimazolam can eliminate concerns about deep extubation. However, it has been suggested that processed electroencephalogram index-guided remimazolam-based anesthesia is occasionally difficult because the index value cannot be maintained within the appropriate ranges even though sufficient doses of remimazolam are administered [4]. This phenomenon may lead to the administration of excessive doses of remimazolam to avoid insufficient sedation. However, large doses of remimazolam can cause prolonged sedation despite its ultrashort duration. In addition, it has been suggested that the residual sedative effect of remimazolam is prolonged in proportion with the length of time for which it is administered [5]. Considering the half-life of flumazenil, it has been noted that resedation even after administration of flumazenil can occur in patients due to overdoses of remimazolam [6]. Taking the abovementioned factors into consideration, both limited use of remimazolam during anesthesia induction if necessary, which may be useful to know its induction dose, and switching to remimazolam-based anesthesia at the end of surgery followed by the administration of flumazenil may be key elements not only to provide easy and safe deep extubation but also to avoid the disadvantages of remimazolam. In addition, we may also advocate use of sevoflurane for anesthesia maintenance in expectation of its bronchodilatory effect in this case [7] because there is no report to identify the bronchodilatory effect of remimazolam. A possible concern may be that the procedural order of deep extubation using remimazolam has not been established. For example, reversal of muscular relaxation after extubation may be an option. We believe that switching to remimazolam followed by administration of flumazenil is one technique to provide easy and safe deep extubation.

## Data Availability

Not applicable.

## References

[1] Daley MD, Norman PH, Coveler LA (1999). Tracheal extubation of adult surgical patients while deeply anesthetized: a survey of United States anesthesiologists. J Clin Anesth.

[2] Juang J, Cordoba M, Ciaramella A, Xiao M, Goldfarb J, Bayter JE, Macias AA (2020). Incidence of airway complications associated with deep extubation in adults. BMC Anesthesiol.

[3] Kim SH, Fechner J (2022). Remimazolam - current knowledge on a new intravenous benzodiazepine anesthetic agent. Korean J Anesthesiol.

[4] Choi BM, Lee JS, Kim KM, Bang JY, Lee EK, Noh GJ (2023). Frequency and characteristics of patients with bispectral index values of 60 or higher during the induction and maintenance of general anesthesia with remimazolam. Sci Rep.

[5] Obara S (2022). Simulation of residual sedation effect of remimazolam: pharmacokinetic-pharmacodynamic simulation can be an additional standard anesthesia monitoring method. J Anesth.

[6] Masui K (2023). Caution!! Reappearance of remimazolam effect after a flumazenil bolus: a larger bolus of flumazenil and a lower total remimazolam clearance are higher risks. J Anesth.

[7] Goff MJ, Arain SR, Ficke DJ, Uhrich TD, Ebert TJ (2000). Absence of bronchodilation during desflurane anesthesia: a comparison to sevoflurane and thiopental. Anesthesiology.

